# Supplementary figures and images for: Ammonification by kelp associated microbes increases ammonium availability
Source: PLoS One. 2024 Mar 29;19(3):e0296622. doi: 10.1371/journal.pone.0296622 (PMC10980195; doi:10.1371/journal.pone.0296622)

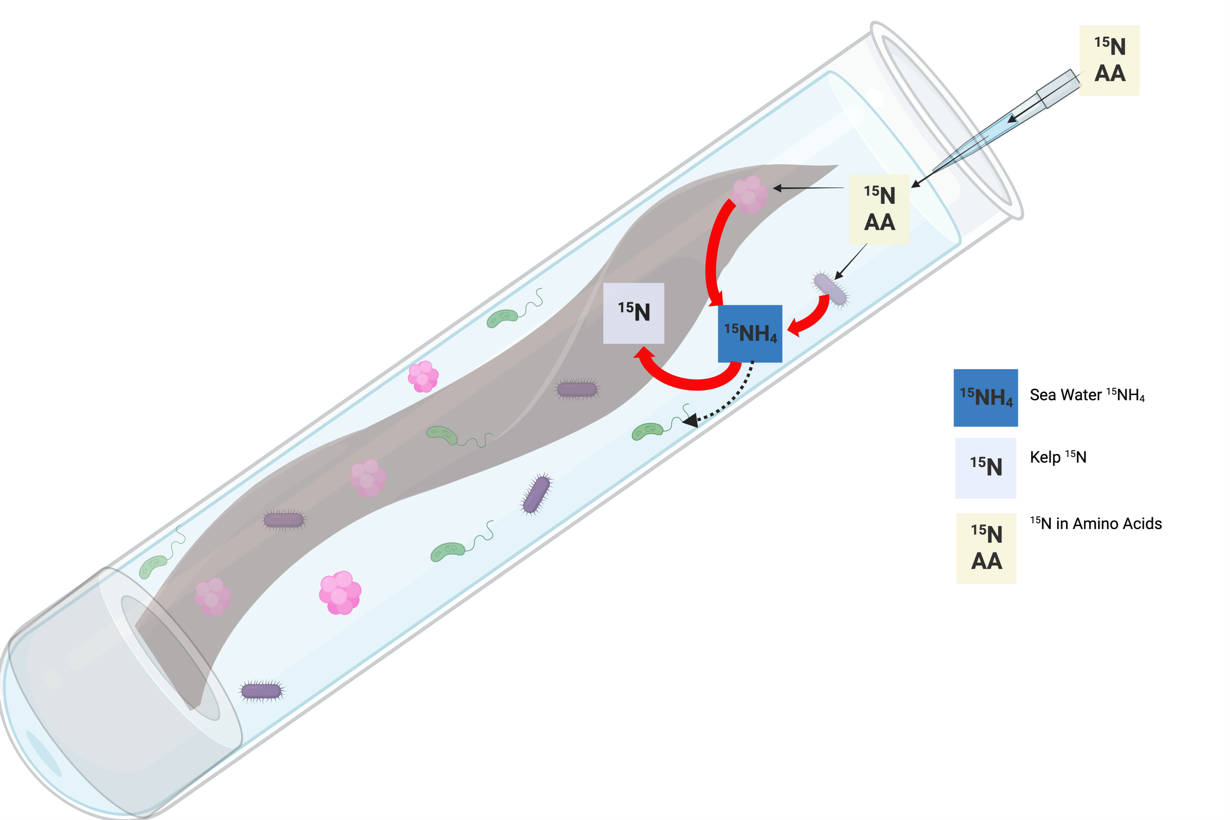

Supplement: S1 Fig — A schematic of the hypothesized movement of enriched 15N in experimental chambers containing Nereocystis when added as 15N amino acids. Red arrows denote those we have quantified, including the microbially-mediated transfer of 15N from amino acids to ammonium via ammonification, a process that could have been done by host-associated or free-living microbes. Chambers without host kelp quantified water column ammonification only. The 15N measured in hosts was assumed to come from this ammonification. The dashed line shows bacterial uptake of ammonium that might have occurred but was not quantified. (TIF) [file pone.0296622.s001.tif]

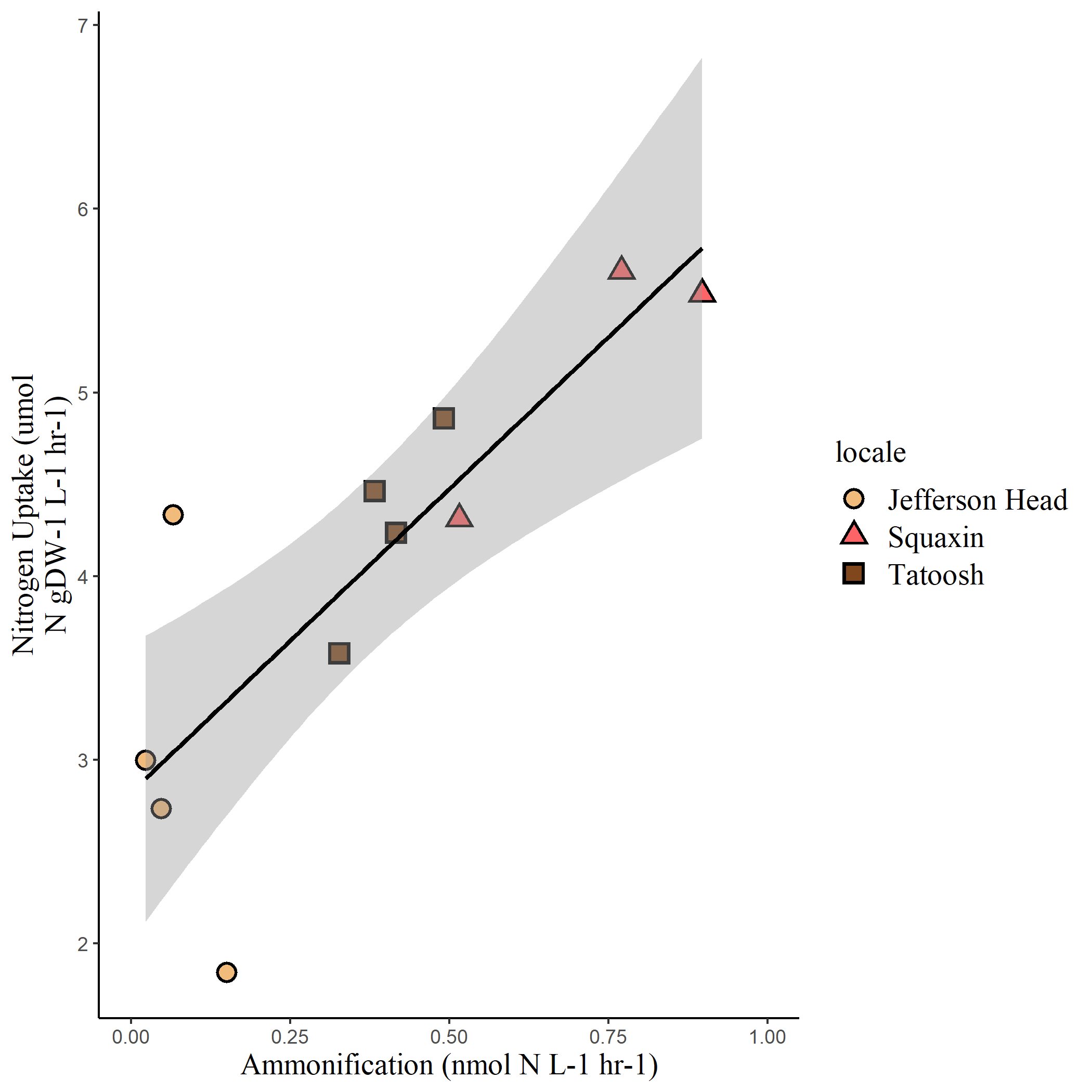

Supplement: S2 Fig — Nitrogen uptake rate (in μmol) vs. ammonification rate (in nmol) for Nereocystis sampling locations. Linear regressions indicated a significant positive association between ammonification and nitrogen Uptake in bull kelp (p = 0.024, r2 = 0.389). The bands around the regression line represents the 95% confidence interval. (TIF) [file pone.0296622.s002.tif]
